# Toxic renal injury at a glance

**DOI:** 10.12861/jrip.2012.07

**Published:** 2012-01-01

**Authors:** Farzad Gheshlaghi

**Affiliations:** ^1^Department of Clinical Toxicology and Forensic Medicine, School of Medicine, Isfahan University of Medical Sciences, Isfahan, Iran.

**Implication for health policy/practice/research/medical:**

Kidneys are particularly susceptible to toxic injury because they receive 20-25% of cardiac output yet make up less than 1% of total body mass.

Kidneys are magic organs because they maintain the constancy of the extracellular fluid by. On the other hand, the kidneys are particularly susceptible to toxic injury because they receive 20-25% of cardiac output yet make up less than 1% of total body mass, they are metabolically active, and thus vulnerable to agents that disrupt metabolism and also they remove water from the filtrate and may build up a high concentration of toxic substances (1,2).

Most nephrotoxicity causes either acute or chronic tubular injury (although glomerular injury may sometimes result from drugs or chemicals) but we can define 3 major syndromes of toxic renal injury: (a) chronic renal failure (CRF) (b) nephrotic syndrome (c) acute renal failure (ARF) (1-3).

Nephrotoxicity of specific substances can be divided in 7 groups:

## 1. Metals

**Barium**, may cause ARF. Barium Inhibits potassium exit from cells, it is likely to cause severe hypokalemia (that unusual in ARF).

**Bismuth**, causes dose-related injury to the proximal tubule dysfunction; in higher doses cause oliguric ARF from tubular necrosis with intestinal inflammation.

**Gold**, generally administered in an organized form, may cause nephrotic syndrome, often with hematuria, which usually reverses over several months after gold is discontinued and chelation instituted. Gold deposits in proximal tubular cells, react to a tubular antigen and the membranous immune complex nephropathy be expected.

**Iron causes**, GI bleeding, vomiting, diarrhea, and shock that causes renal ischemia and ARF.

**Lead**, inhibits sulphydryl-dependent enzymes and slowly injures proximal tubular cells, causing chronic interstitial nephritis with fibrosis, exacerbated by hypertension.

**Mercury**, has a strong affinity for renal tissue, especially as mercuric ion, although organic mercury and elemental mercury vapor are also nephrotoxic. Mercury necroses proximal tubular cells. Oliguric acute renal failure with tubular cell casts is typical.

**Thallium**, poisoning is associated with loss of urinary concentrating ability and albuminuria and sometimes acute renal failure with tubular necrosis and interstitial inflammation.

**Lithium**, commonly causes polyuria without pathologic changes. Some patients develop distal renal tubular acidosis. There are occasional reports of both acute renal failure and nephrotic syndrome associated with lithium therapy (1,2).

## 2. Solvents

**Carbon tetrachloride (CCl**_4_ **)**, known for its hepatotoxicity, is also a nephrotoxin via the hepatorenal syndrome or via direct renal tubular injury (necrosis of the proximal tubule and loop of Henle). CCl_4_ is converted to trichloromethyl and trichloromethyl peroxyl free radicals (by P450 enzymes in proximal tubules). These free radicals are the probable causes of cell necrosis. The lesion is usually reversible if the patients recovers from the hepatic injury (1,2).

**Tetrachloroethylene**, poisoning resembles that of CCl_4 _, with hepatotoxicity predominating occasionally acute renal failure from tubular necrosis.

**Toluene** causes hipporic acidosis, high anioin gap acidosis. A distal renal tubular acidosis, severe hypokalemia (1,2).

## 3. Glycols

Ethylene glycol is itself nonnephrotoxic, hepatically metabolized to glycolic acid and then oxalic acid. Deposition of calcium oxalate crystals in the tubules causes obstruction and acute renal failure. Crystals also provoke severe interstitial inflammation causes hematuria and proteinuria, followed by oliguria or anuria (1-3).

## 4. Non-steroidal anti-inflammatory drugs (NSAIDs)

Incidence of NSAIDs nephropathy is lower than GI side effect but with such high usage rate (several months), we see the renal side effect. 4 main nephrotoxicity “syndromes” due to NSAIDs are

1. Interstitial nephritis (most common renal dis.)
2 .pre-renal/renal ARF
3. K+ retention
4. Hypertension (3,4).

## 5. Others

**Anesthetic Agent** generally decrees blood pressure and causes ARF.

Fluorinated agent (Methoxyflurane (most), Halothane (much less), Enflurane (rarely) are directly Nephrotoxic due to production of fluoride (1-4).

## Herbicides

**Paraquat:** one of the organ which involved in paraquat intoxication is kidney but fortunately this renal failure is fully reversible if the patient survived (1,2).

## 6. Drug-Abuse Nephropathy

Contaminant drugs and infection in substances abuser causes CRF, nephrotic syndrome.

LSD causes retroperitoneal fibrosis in long term usage.

Amphetamines by polyvasculitis mechanism induce CRF (1-3).

## 7. Pigment Nephropathy

**Myoglobinuria:** There are many substances causes myoglobinuria by different mechanism, like alcohol, diuretics, antipsychotics, anticholinergic, opioids, sedative, cocaine, theophylline, CO poisoning, strychnine (1).

## Author’s contribution

FG is the single author of the manuscript.

## Conflict of interests

The author declared no competing interests.

## Ethical considerations

Ethical issues (including plagiarism, data fabrication, double publication) have been completely observed by the author.

## Funding/Support

None.
